# Transcriptional Suppression of CPI-17 Gene Expression in Vascular Smooth Muscle Cells by Tumor Necrosis Factor, Krüppel-Like Factor 4, and Sp1 Is Associated with Lipopolysaccharide-Induced Vascular Hypocontractility, Hypotension, and Mortality

**DOI:** 10.1128/MCB.00070-19

**Published:** 2019-05-14

**Authors:** Guogang Zhao, Yu Zhong, Wen Su, Shu Liu, Xiulong Song, Tianfei Hou, Xufang Mu, Ming Cui Gong, Zhenheng Guo

**Affiliations:** a Saha Cardiovascular Research Center, University of Kentucky, Lexington, Kentucky, USA; b Department of Pharmacology and Nutritional Science, University of Kentucky, Lexington, Kentucky, USA; c College of Life Science, Hebei Agricultural University, Baoding, Hebei Province, China; d Department of Physiology, College of Medicine, University of Kentucky, Lexington, Kentucky, USA; e Research and Development, Lexington Veterans Affairs Medical Center, Lexington, Kentucky, USA

**Keywords:** CPI-17, hypotension, KLF4, LPS, sepsis, Sp1, vascular hypocontractility, vasodilatory shock, transcriptional regulation

## Abstract

Vasodilatory shock in sepsis is caused by the failure of the vasculature to respond to vasopressors, which results in hypotension, multiorgan failure, and ultimately patient death. Recently, it was reported that CPI-17, a key player in the regulation of smooth muscle contraction, was downregulated by lipopolysaccharide (LPS) in mesenteric arteries concordant with vascular hypocontractilty. While Sp1 has been shown to activate CPI-17 transcription, it is unknown whether Sp1 is involved in LPS-induced smooth muscle CPI-17 downregulation. Here we report that tumor necrosis factor (TNF) was critical for LPS-induced smooth muscle CPI-17 downregulation. Mechanistically, we identified two GC boxes as a key TNF response element in the CPI-17 promoter and demonstrated that KLF4 was upregulated by TNF, competed with Sp1 for the binding to the GC boxes in the CPI-17 promoter, and repressed CPI-17 transcription through histone deacetylases (HDACs). Moreover, genetic deletion of TNF or pharmacological inhibition of HDACs protected mice from LPS-induced smooth muscle CPI-17 downregulation, vascular hypocontractility, hypotension, and mortality. In summary, these data provide a novel mechanism of the transcriptional control of CPI-17 in vascular smooth muscle cells under inflammatory conditions and suggest a new potential therapeutic strategy for the treatment of vasodilatory shock in sepsis.

## Abstract (precis)

Vasodilatory shock in sepsis is caused by the failure of the vasculature to respond to vasopressors, which results in hypotension, multiorgan failure, and ultimately patient death. Recently, it was reported that CPI-17, a key player in the regulation of smooth muscle contraction, was downregulated by lipopolysaccharide (LPS) in mesenteric arteries concordant with vascular hypocontractilty.

## INTRODUCTION

Sepsis is a life-threatening disease with a high mortality rate of up to 50% affecting over 750,000 patients in the United States (1). Sepsis is defined as a severe inflammatory response to infection, and its complications, including hypotension, can be fatal (2). Thus, one of the most critical guidelines for clinical management of sepsis is the treatment of patients with vasopressors to elevate blood pressure (BP) and maintain organ perfusion (3). Unfortunately, many patients with sepsis respond poorly to vasopressors and exhibit a vasodilatory shock as characterized by persistent and irreversible hypocontractility and hypotension (2), which leads to multiorgan failure and ultimately patient death (2).

Several potential mechanisms have been proposed for vasodilatory shock: (i) excessive production of nitric oxide (NO) by induction of inducible nitric oxide synthase (iNOS), (ii) increased levels of prostacyclin by cyclooxygenase 2, (iii) elevated peroxynitrite by superoxide, (iv) activation of vascular ATP-sensitive and calcium-activated potassium channels, and (v) deficiency in corticosteroid and vasopressin (2, 4). However, the clinical trials based on these potential mechanisms have led to disappointing results (4, 5). Thus, there is an urgent need to explore additional mechanisms that may lead to the development of a new therapeutic strategy against this severe disease.

Accumulating evidence suggests that dysfunction of peripheral arterial vascular smooth muscle cells (VSMCs) may also play a pivotal role in vasodilatory shock. Progress made in the last decades has established a smooth muscle contractile machinery composed of a contractile unit (smooth muscle myosin and actin) and regulatory proteins, including myosin light chain kinase (MLCK), protein kinase C (PKC), small GTPase Rho-A, Rho kinase (ROCK), myosin phosphatase targeting subunit 1 (MYPT1), and protein kinase C-potentiated phosphatase inhibitor of 17 kDa (CPI-17) (6, 7). Among them, CPI-17 is particularly interesting. CPI-17 is expressed predominantly in VSMCs and has been demonstrated to play a crucial role in the regulation of smooth muscle contraction and relaxation and BP homeostasis (7–9). Consistent with its physiological role, CPI-17 is implicated in the etiology of many human diseases, including asthma, diabetic vascular complication, ischemia, hypoxia, cancer, vascular injury, hypertension, and inflammatory bowel diseases (9–16). In line with these findings, Reho et al. recently reported that CPI-17 protein was downregulated in mesenteric arteries concordant with reduced force generation to depolarization, Ca^2+^ increase, and phenylephrine (PE) in a mouse lipopolysaccharide (LPS) model of sepsis (17), indicating that downregulation of smooth muscle CPI-17 may play a role in vasodilatory shock.

There are limited studies on the regulation of CPI-17 at the transcriptional level, which is in sharp contrast to its posttranslational modifications (e.g., phosphorylation) that has been extensively studied (7). Boopathi et al. identified the binding sites for transcription factor GATA-6 and nuclear factor kappa B (NF-κB) in a mouse CPI-17 promoter and demonstrated that GATA-6 and NF-κB overexpression was associated with CPI-17 overexpression in bladder smooth muscle from men with bladder hypertrophy and a mouse model of bladder outlet obstruction (10). More recently, Kim et al. identified the binding sites (GC boxes) for transcription factor Sp1 in the mouse and human CPI-17 promoters and demonstrated that Sp1 activates CPI-17 transcription in rat aortic VSMCs (18). However, whether GATA-6, NF-κB, and Sp1 are involved in CPI-17 downregulation in VSMCs in vasodilatory shock is unknown.

Here we report that vascular hypocontractility and hypotension were associated with a selective CPI-17 downregulation in mesenteric arteries from mice injected with LPS. We further demonstrated that tumor necrosis factor alpha (TNF), a pleiotropic inflammatory cytokine, mimicked LPS in downregulation of CPI-17 in VSMCs *in vivo* (mice), *ex vivo* (organ culture), and *in vitro* (cell culture). Moreover, we identified the two proximal GC boxes in the CPI-17 promoter as a key *cis* TNF response element critical for TNF-induced CPI-17 transcriptional suppression. Mechanistically, we demonstrated that the transcription factor Krüppel-like factor 4 (KLF4) competed with Sp1 for binding to the same GC boxes in the CPI-17 promoter thus suppressed Sp1-mediated CPI-17 transcription via histone deacetylases (HDACs). Finally, we showed that genetic deletion of TNF or pharmacological inhibition of HDAC protected mice from LPS-induced CPI-17 downregulation, vascular hypocontractility, hypotension, and mortality.

## RESULTS

### Vascular hypocontractility and hypotension are associated with selective CPI-17 downregulation in mesenteric arteries from mice injected with LPS.

**FIG 1:**
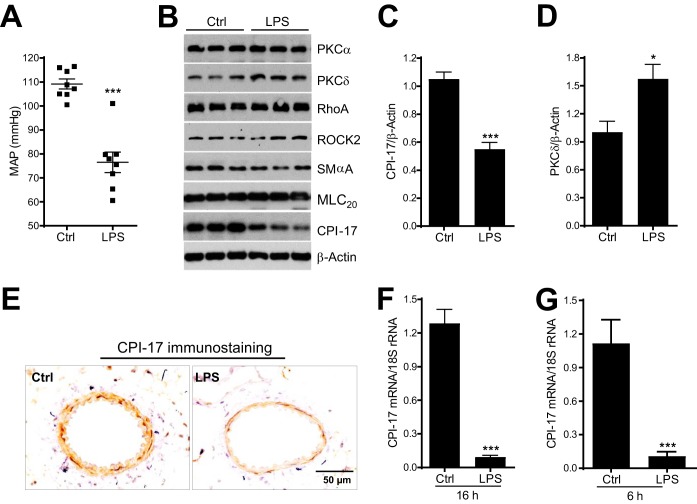
LPS-induced hypotension is associated with selective CPI-17 downregulation in mesenteric arterial smooth muscle cells. (A) Mean arterial pressure (MAP) in mice injected with LPS or saline (Ctrl) for 16 h. (B) Representative immunoblots of PKCα, PKCδ, RhoA, ROCK2, SMαA, MLC_20_, CPI-17, and β-actin protein expression in mesenteric arteries from mice 16 h after LPS or saline injection. (C and D) Semiquantitative analysis of immunoblots of CPI-17 and PKCδ protein expression (*n* = 5 to 9). (E) Representative immunohistochemical staining of CPI-17 protein expression in cross sections of paraffin-embedded mesenteric arteries from mice 16 h after LPS or saline injection (*n* = 3). (F and G) Quantitative PCR analysis of CPI-17 mRNA expression in mesenteric arteries from mice 6 or 16 h after LPS or saline injection (*n* = 3 to 6). *, *P < *0.05; ***, *P *< 0.001 versus Ctrl.

To study vasodilator shock in sepsis, C57BL/6 mice were injected intravenously with LPS (10 mg/kg of body weight). The effect of LPS on BP was determined by telemetry. Consistent with that in patients with sepsis (2), hypotension was found in mice 16 h after LPS injection compared to BP before LPS injection (control [Ctrl]) (Fig. 1A). The effect of LPS on vascular smooth muscle contractile response to vasopressors was determined in mesenteric arteries strips from mice 16 h after LPS injection. Consistent with the previous reports (17, 19), a reduced vascular smooth muscle contractile response to PE or serotonin (5-HT) was found in mesenteric arteries from mice injected with LPS compared to that in mice injected with saline (data not shown).

To define the mechanism that underlies LPS-induced vasodilatory shock, mesenteric arteries were isolated from mice 16 h after LPS injection and then subjected to Western blot analysis with a panel of antibodies specific for the proteins that have established roles in agonist-induced smooth muscle contraction (6). Interestingly, of all the proteins that we examined, including PKC alpha (PKCα), PKC delta (PKCδ), RhoA, ROCK2, smooth muscle α-actin (SMαA), 20-kDa myosin light chain (MLC_20_), and CPI-17, CPI-17 was selectively downregulated by LPS relative to that in the control mice injected with saline (Fig. 1B and C). In contrast, other proteins either showed no change (e.g., RhoA [Fig. 1B]) or were upregulated by LPS relative to the levels in control mice (e.g., PKCδ [Fig. 1B and D]).

To determine whether LPS-induced CPI-17 downregulation in mesenteric arteries occurs in VSMCs, we performed CPI-17 immunostaining studies in paraffin-embedded mesenteric artery cross sections from mice 16 h after LPS injection. Consistent with the result of Western blotting, diminished CPI-17 protein immunostaining was observed in the medial VSMC layer of mesenteric arteries from mice injected with LPS compared to that in control mice injected with saline (Fig. 1E).

To investigate whether CPI-17 mRNA is affected by LPS, we determined the relative abundance of CPI-17 mRNA by real-time PCR in mesenteric arteries from mice 16 h after LPS injection. Consistent with CPI-17 protein downregulation, CPI-17 mRNA was reduced by more than 90% in mesenteric arteries from mice 16 h after LPS injection compared to that in control mice injected with saline (Fig. 1F). We also determined CPI-17 mRNA expression in mesenteric arteries from mice 6 h after LPS injection and found a reduction of CPI-17 mRNA similar to that in mice 16 h after LPS injection (Fig. 1G).

### TNF is markedly upregulated by LPS in mesenteric arteries and is sufficient to induce hypotension and CPI-17 downregulation *in vivo* (in mice), *ex vivo* (organ culture), and *in vitro* (VSMC culture).

To investigate the mechanism by which LPS downregulates CPI-17 in mesenteric arteries, we explored the possibility that LPS induces CPI-17 downregulation through TNF. We focused on TNF not only because it is well established that TNF is upregulated by LPS (20) but also because it was recently reported that TNF is responsible for CPI-17 downregulation in visceral smooth muscle tissues in inflammation animal models (11).

**FIG 2:**
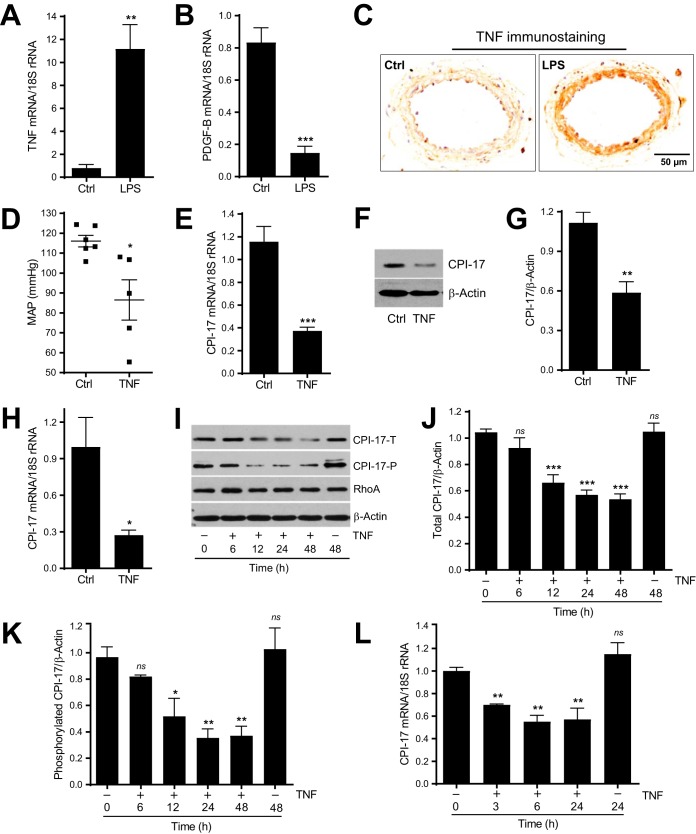
TNF is upregulated by LPS in mesenteric arteries and is sufficient to induce hypotension and CPI-17 downregulation in vascular smooth muscle cells *in vitro* and *in vivo*. (A and B) Quantitative PCR analysis of TNF and PDGF-B mRNA expression in mesenteric arteries from mice 6 h after LPS or saline injection (*n* = 3 to 6). (C) Representative immunohistochemical staining of TNF protein expression in cross sections of paraffin-embedded mesenteric arteries from mice 16 h after LPS or saline injection (*n* = 3). (D) Mean arterial pressure (MAP) in mice injected with TNF or saline for 6 h. (E) Quantitative PCR analysis of CPI-17 mRNA expressions in mesenteric arteries from mice 6 h after TNF or saline injection (*n* = 6). (F) Representative immunoblots of CPI-17 and β-actin protein expression in mouse aortas in organ culture in the presence of TNF (10 ng/ml) or saline for 48 h. (G) Semiquantitative analysis of immunoblots of CPI-17 expression (*n* = 4 to 6). (H) Quantitative PCR analysis of CPI-17 mRNA expression in mouse aortas in organ culture in the presence of TNF (10 ng/ml) or saline (Ctrl) for 48 h (*n* = 5). (I) Representative immunoblot of total CPI-17 (CPI-17-T), phosphorylated CPI-17 (CPI-17-P), RhoA, and β-actin protein expression in cultured rat aortic VSMCs incubated with TNF (10 ng/ml) or saline for the time indicated. (J and K) Semiquantitative analysis of immunoblots of total CPI-17 (*n* = 6 or 7) and phosphorylated CPI-17 (*n* = 3) protein expression. (L) Quantitative PCR analysis of CPI-17 mRNA expression in cultured rat aortic VSMCs treated with TNF (10 ng/ml) or saline (*n* = 4) for the time indicated. *, *P < *0.05; **, *P < *0.01; ***, *P < *0.001; ns, not significant versus Ctrl (A, B, D, E, G, and H) or absence of TNF at time zero (J, K, and L).

Several distinct approaches were taken to robustly investigate whether TNF is involved in LPS-induced CPI-17 downregulation. First, we determined whether TNF mRNA is upregulated by LPS in mesenteric arteries from mice 6 h after LPS injection. In contrast to a remarkable CPI-17 mRNA downregulation as shown in Fig. 1G, a dramatic TNF mRNA upregulation was observed in the same set of mesenteric artery RNA preparations from mice injected with LPS compared to that in control mice injected with saline (Fig. 2A). We also determined platelet-derived growth factor subunit B (PDGF-B) mRNA expression in the same set of mesenteric artery RNA preparations because it has been shown that PDGF inhibited CPI-17 mRNA in cultured VSMCs (18). Surprisingly, PDGF-B mRNA, in contrast to TNF mRNA, was downregulated in mesenteric arteries from mice injected with LPS compared to that in control mice injected with saline (Fig. 2B versus Fig. 2A).

Second, to investigate whether LPS-induced TNF mRNA upregulation is translated into TNF protein upregulation, we performed TNF immunostaining studies in the same set of paraffin-embedded mesenteric artery cross sections as that in CPI-17 immunostaining studies (Fig. 1E). As shown in Fig. 2C, a robust increase in TNF immunostaining was observed in the medial VSMC layer of mesenteric arteries from mice 16 h after LPS injection compared to that in control mice injected with saline. Of note, there was a spatial correlation between TNF protein upregulation and CPI-17 downregulation in mesenteric arteries from mice 16 h after LPS injection (Fig. 2C versus Fig. 1E).

Third, to investigate whether upregulation of TNF by LPS affects BP and CPI-17 transcription, C57BL/6 mice were injected intravenously with TNF (6 μg per mice) to induce vasodilatory shock as previously described (21). Consistent with the effect of LPS on BP, TNF also induced hypotension in mice 6 h after TNF injection compared to that in control mice before TNF injection (Fig. 2D). Similar to the effect of LPS on CPI-17 mRNA expression, TNF also downregulated CPI-17 mRNA in mesenteric arteries 6 h after TNF injection compared to that in control mice injected with saline (Fig. 2E).

Fourth, to investigate whether TNF can directly induce CPI-17 downregulation, aortas were isolated from C57BL/6 mice and cultured in Krebs solution with or without TNF (10 ng/ml) for 48 h. We chose 10 ng/ml of TNF in aortic organ culture studies because the similar concentration of TNF has been reported in serum from mice 1.5 h after LPS injection (22). Consistent with the effect of TNF on CPI-17 protein expression in ileal organ culture (11), CPI-17 protein (Fig. 2F and G) and mRNA (Fig. 2H) were downregulated in aortas treated with TNF compared to the levels in control aortas without TNF treatment.

Fifth, to define the mechanism by which TNF downregulates CPI-17, we sought to establish a cell culture system to mimic downregulation of CPI-17 by LPS or TNF *in vivo*. Primary rat aortic VSMCs were starved in fetal bovine serum (FBS)-free medium for 24 h and then treated with TNF (10 ng/ml) in fresh FBS-free Dulbecco modified Eagle medium (DMEM) for 6, 12, 24, and 48 h. The total CPI-17 (CPI-17-T) and phosphorylated CPI-17 (CPI-17-P) expressions were determined by Western blotting using antibodies specific for CPI-17-T and CPI-17-P (T38) as previously described (13, 23, 24). Representative immunoblots (Fig. 2I) and quantitative data (Fig. 2J and K) demonstrated that CPI-17-T and CPI-17-P, but not RhoA, were downregulated in the cells treated by TNF in a time-dependent manner compared to that in control cells (the cells at time zero; time zero was defined as the onset of TNF treatment). To rule out the potential effect of the timing of cell culture on TNF-induced CPI-17 downregulation, CPI-17-T and CPI-17-P were also determined in cells cultured for 48 h in the absence of TNF. As shown in Fig. 2I to K, there was no significant difference in CPI-17-T and CPI-17-P levels between cells at time zero and at 48 h without TNF, indicating that TNF, but not the timing of cell culture, is responsible for the observed CPI-17 downregulation.

Finally, to investigate whether, in cultured VSMCs, TNF-induced CPI-17 downregulation occurs at a transcriptional level, serum-starved rat aortic VSMCs were treated or not treated with TNF (10 ng/ml) for 3, 6, and 24 h. The effect of TNF on CPI-17 mRNA expression was determined by real-time PCR. As shown in Fig. 2L, TNF inhibited CPI-17 mRNA expression in a time-dependent manner compared to that in control cells (the cells at time zero). Of note, there was also no difference in CPI-17 mRNA levels between cells at time zero and at 48 h without TNF.

### Identification of the two proximal GC boxes in the CPI-17 promoter as a key *cis* TNF response element critical for TNF-induced CPI-17 transcriptional suppression.

To investigate the mechanism that underlies TNF-induced CPI-17 transcriptional suppression, we cloned an 817-bp CPI-17 promoter from a mouse BAC clone corresponding to the 5′-flanking sequence of the CPI-17 gene from bp −792 to +25 relative to the transcription start site (designated +1). To identify a key *cis* TNF response element critical for TNF-induced CPI-17 transcriptional suppression, a series of 5′-end-truncated CPI-17 promoter-luciferase constructs were generated by PCR cloning and were transfected with a pRL *Renilla* luciferase control vector into rat aortic VSMCs treated or not treated with TNF (10 ng/ml, 48 h). The effect of TNF on CPI-17 promoter activity was determined by a modified dual-luciferase assay (25, 26).

**FIG 3:**
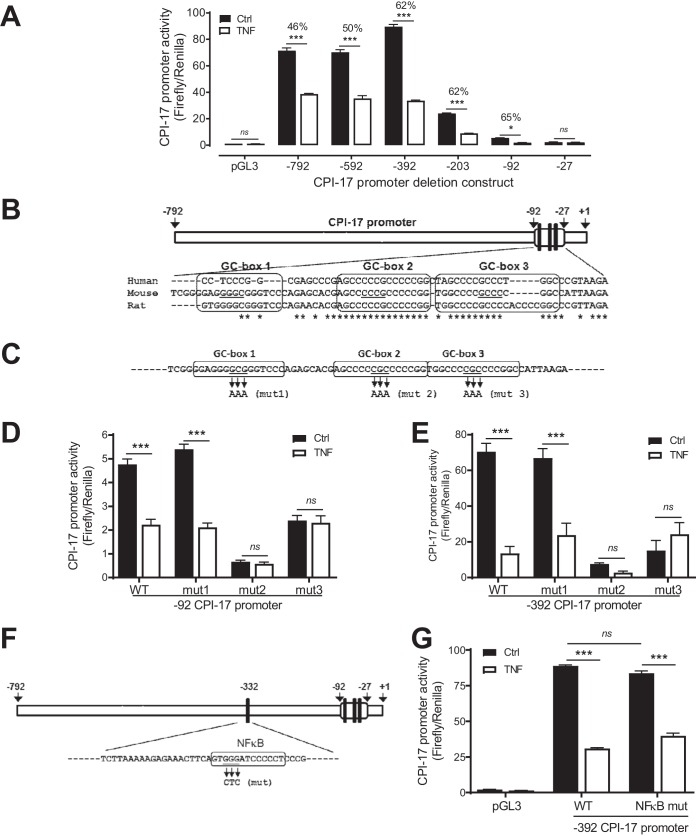
Identification of two proximal GC boxes in the CPI-17 promoter as a key *cis* TNF response element critical for TNF to suppress CPI-17 transcription in VSMCs. (A) A series of 5′ deletions from bp −792 to −27 (relative to the transcriptional start site) of CPI-17 promoter-firefly luciferase constructs were cotransfected with a *Renilla* luciferase control vector into rat aortic VSMCs. CPI-17 promoter activity was determined 48 h after TNF (10 ng/ml) or saline (Ctrl) treatment (*n* = 6). The percentage of TNF-induced transcriptional suppressions is indicated for each construct. (B) Schematic diagram of the predicted CPI-17 promoter and three proximal GC boxes in the CPI-17 promoter. The sequences of three proximal GC boxes in the CPI-17 promoter are enclosed. The core sequences of GC boxes are underlined. (C) Schematic diagram of three core nucleotide acids in three GC boxes that were subjected to site-directed mutagenesis. (D and E) Analysis of CPI-17 promoter activity in rat aortic VSMCs transfected with wild-type (WT) or mutant (mut) bp −92 (D) or −392 (E) constructs and treated with TNF (10 ng/ml) or saline for 48 h (*n* = 6 to 18). (F) Schematic diagram of an NF-κB binding site in the CPI-17 promoter. The core sequences of the NF-κB binding site in the CPI-17 promoter are enclosed, and three core nucleotide acids in the NF-κB binding site that were subjected to site-directed mutagenesis are underlined. (G) Analysis of CPI-17 promoter activity in rat aortic VSMCs transfected with WT or mut bp −392 constructs and treated with TNF (10 ng/ml) or saline for 48 h (*n* = 3 to 12). *, *P < *0.05; ***, *P < *0.001.

In the absence of TNF, the bp −792 construct exhibited a promoter activity 115-fold over that achieved by the pGL3-basic vector (Fig. 3A). Progressive 5′ truncations of the bp −792 construct from bp −792 to −392 had little effect on promoter activity, but further progressive truncations from bp −392 to −27 resulted in a substantial and progressive diminution of promoter activity (Fig. 3A). These results suggest that a 366-bp region between bp −392 and −27 in the CPI-17 promoter is vital for its basal promoter activity.

In the presence of TNF, the bp −792 promoter activity was decreased by 46% (Fig. 3A). Progressive 5′ truncations of the bp −792 promoter construct from bp −792 to −92 had little effect on the CPI-17 promoter activity in responses to TNF despite having a profound effect on basal CPI-17 promoter activity (from bp −392 to −92). Further truncation from bp −92 to −27 completely abolished TNF-induced CPI-17 transcriptional suppression. These results suggest that a 66-bp region between bp −92 and −27 in the proximal CPI-17 promoter contains a *cis* TNF response element critical for TNF to suppress CPI-17 transcription.

To identify transcription factors that respond to TNF to bind to the *cis* TNF response element in the CPI-17 promoter, the bp −792 promoter was analyzed by Genomatix MatInspector to search potential transcription factor binding sites. The resultant analysis predicted 160 putative transcription factor binding sites in the bp −792 promoter (data not shown), including the three GC boxes located between bp −92 and −27 of the CPI-17 promoter (Fig. 3B). Of the three GC boxes, GC boxes 2 and 3 were highly conserved among mouse, rat, and human sequences (Fig. 3B), indicating that GC boxes 2 and 3 may be functionally more important than GC box 1 in CPI-17 transcription.

To determine which proximal GC box in the CPI-17 promoter is critical to TNF-induced CPI-17 transcriptional suppression, the core sequence of each GC box (GCG or CGC) in the bp −92 construct was changed to adenine by site-directed mutagenesis (Fig. 3C). The wild-type (WT) and mutant (mut) CPI-17 promoter activity was determined in rat aortic VSMCs treated with TNF (10 ng/ml) for 48 h. Consistent with the level of conserved nucleic acid sequences in three GC boxes (Fig. 3B), mutation of GC box 2 or 3, but not GC box 1, suppressed the basal CPI-17 transcription and resulted in an elimination of TNF-induced CPI-17 transcriptional suppression (Fig. 3D).

It was noted that the basal promoter activity of the bp −92 construct was considerably lower than that of less truncated constructs (Fig. 3A). To verify that the results from the bp −92 construct holds for a longer construct, we performed the same site-directed mutagenesis and promoter assay in the bp −392 construct that had the highest basal CPI-17 promoter activity (Fig. 3A). Again, mutations of GC boxes 2 and 3, but not GC box 1, suppressed the basal CPI-17 transcription and abolished TNF-induced CPI-17 transcriptional suppression (Fig. 3E).

Genomatix MatInspector also predicted a proximal NF-κB binding site in the CPI-17 promoter (Fig. 3F). NF-κB is recently implicated in the regulation of CPI-17 transcription in cultured human and mouse bladder smooth muscle cells (10). Therefore, we wondered whether this putative NF-κB binding site is involved in TNF-induced CPI-17 transcriptional suppression. Surprisingly, mutation of the core sequence of NF-κB binding site in the bp −392 construct (Fig. 3F) that has been shown to be able to abolish NF-κB DNA binding activity (27) affected neither basal CPI-17 transcription nor TNF-induced CPI-17 transcriptional suppression (Fig. 3G).

### TNF suppresses transcription of the CPI-17 gene through inhibiting Sp1 binding to the proximal GC boxes in the CPI-17 promoter.

**FIG 4:**
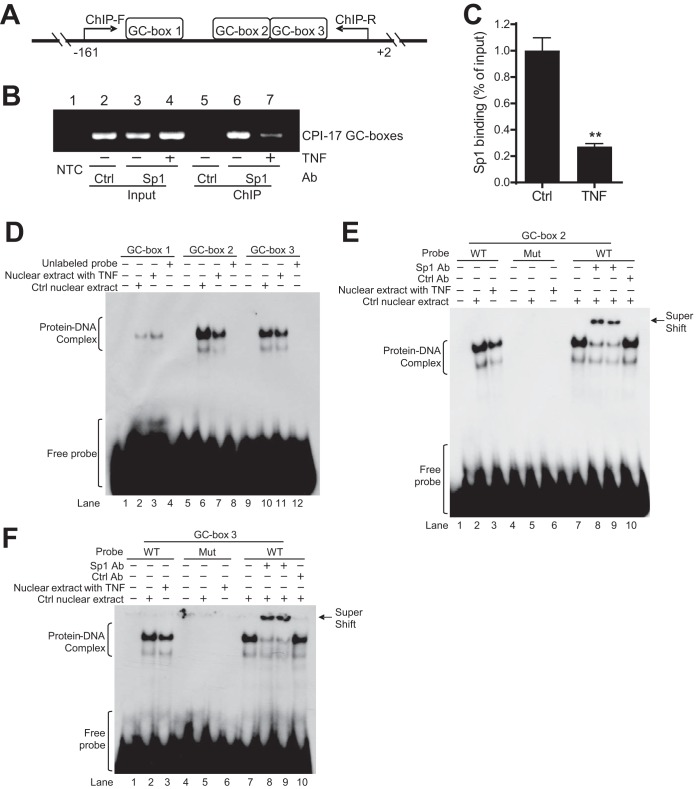
TNF inhibits Sp1 binding to proximal GC boxes in the CPI-17 promoter in VSMCs. (A) Schematic diagram of ChIP-PCR for analysis of the binding of Sp1 to proximal GC boxes in the CPI-17 promoter. ChIP-F, forward primers; ChIP-R, reverse primers. (B and C) Representative ChIP-PCR (B) and semiquantitative data (C; *n* = 4) showing a diminished Sp1 binding to GC boxes in cultured mouse aortic VSMCs treated with TNF (10 ng/ml, 48 h) relative to that in control cells. NTC, no template control to ensure ChIP-PCR specificity. Ctrl Ab, a nonspecific control antibody to ensure Sp1 antibody specificity. (D) Representative EMSA showing a reduced protein-DNA complex formation with GC boxes 2 and 3, but not GC box 1, in nuclear extracts from cultured mouse aortic VSMCs treated with TNF relative to that in control cells (*n* = 3). (E and F) Representative EMSA showing the binding of Sp1 to WT GC box 2 (E) and GC box 3 (F) but not mutant GC boxes (*n* = 3). ***, *P* < 0.001 versus Ctrl.

It has long been known that Sp1 can bind to GC boxes in many gene promoters to regulate gene expressions in a promoter-, cell-, and, tissue-specific manner (28). In particular, it was recently reported that Sp1 binds to the proximal GC boxes in the CPI-17 promoter and activates CPI-17 transcription in cultured rat aortic VSMCs (18). To investigate whether the binding of Sp1 to the proximal GC boxes in the CPI-17 promoter is involved in TNF-induced CPI-17 transcriptional suppression, we performed chromatin immunoprecipitation (ChIP) assays in mouse aortic VSMCs treated or not treated with TNF (10 ng/ml, 48 h) to determine whether the binding of Sp1 to GC boxes in the proximal CPI-17 promoter is affected by TNF (Fig. 4A).

Consistent with the previous report (18), Sp1 bound to the proximal GC boxes in the CPI-17 promoter in mouse aortic VSMCs in the absence of TNF (Fig. 4B, lane 6). The observed Sp1 binding was specific because there was no Sp1 binding in the cells with NTC (no template control to ensure the specificity of the PCR [Fig. 4B, lane 1]) or the cells with the control antibody (nonspecific rabbit IgG to ensure the specificity of Sp1 antibody [Fig. 4B, lane 5]). Moreover, the binding of Sp1 to the proximal GC boxes in the CPI-17 promoter was markedly diminished in the cells treated with TNF compared to that in control cells without TNF treatment (Fig. 4B, lane 6 versus lane 7). The ChIP-PCR products were normalized to the corresponding input and were semiquantified by NIH ImageJ software as described previously (25). The semiquantitative data showed that the binding of Sp1 to the proximal GC boxes in the CPI-17 promoter was reduced by 72% in the cells treated with TNF compared to that in control cells without TNF treatment (Fig. 4C).

To define which proximal GC box in the CPI-17 promoter is bound by Sp1 and is critical for TNF-induced CPI-17 transcriptional suppression, nuclear extracts from rat aortic VSMCs treated or not treated with TNF (10 ng/ml, 48 h) were incubated with ^32^P-labeled probes containing GC box 1, 2, or 3 to form protein-DNA complexes. In the absence of TNF, nuclear extracts bound to all three probes to form protein-DNA complexes (Fig. 4D, lanes 2, 6, and 10), but the yields of the protein-DNA complexes were very different: many more protein-DNA complexes were found with probe 2 or 3 than with probe 1 (Fig. 4D, lane 2 versus lane 6 or 10). The observed protein-DNA complexes were specific because no protein-DNA complexes were observed in the absence of nuclear protein (Fig. 4D, lanes 1, 5, and 9) or the presence of excessive unlabeled (“cold”) probes as competitors (Fig. 4D, lanes 4, 8, and 12). Interestingly, in the presence of TNF, the observed protein-DNA complexes were diminished with probe 2 or 3, but not with probe 1, relative to that in the absence of TNF (Fig. 4D, lane 2 versus lane 3, lane 6 versus lane 7, and lane 10 versus lane 11).

To determine whether the DNA-protein complexes with probe 2 or 3 contain Sp1, nuclear extracts from rat aortic VSMCs were preincubated with an antibody specific for Sp1 or a control antibody before incubation with ^32^P-labeled probe 2 or 3. Compared to the controls without antibody addition (Fig. 4E and F, lanes 7), preincubation of nuclear extracts with the Sp1 antibody, but not the control antibody, induced a clear supershifted band (Fig. 4E and F, lanes 8 or 9 versus lanes 10). These results demonstrated that the DNA-protein complexes with probe 2 or 3 indeed contain Sp1.

The results of site-directed mutagenesis studies demonstrated that GC box 2 or 3, but not GC box 1, is critical for basal CPI-17 transcription and TNF-induced CPI-17 transcriptional suppression (Fig. 3C to E). However, it remains elusive whether mutation of GC box 2 or 3 affects Sp1 binding to GC box 2 or 3 and thereby affects basal CPI-17 transcription and TNF-induced CPI-17 transcriptional suppression. To address this mechanistic issue, nuclear extracts were incubated with mutant ^32^P-labeled probe 2 or 3 containing the same GC box mutations as that in Fig. 3C to E. The results showed that mutation of GC box 2 or 3 abolished protein-DNA complex formation (Fig. 4E and F, lanes 5 and 6 versus lanes 2 and 3).

**FIG 5:**
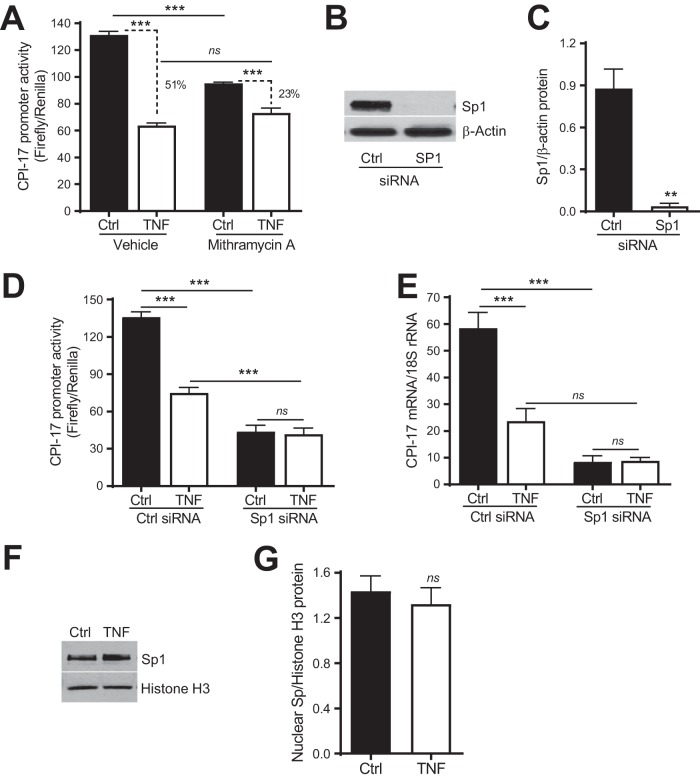
SP1 is required for TNF to suppress CPI-17 transcription in VSMCs. (A) Analysis of CPI-17 promoter activity in rat aortic VSMCs treated with Sp1 antagonist mithramycin A (50 nM) or a vehicle (DMSO) for 48 h (*n* = 6). (B) Representative immunoblots of Sp1 and β-actin protein expression in rat aortic VSMCs transfected with Sp1 siRNA (1 nM) or a scrambled siRNA (Ctrl) for 48 h. (C) Semiquantitative analysis of Sp1 immunoblots (*n* = 3). (D) Analysis of CPI-17 promoter activity in rat aortic VSMCs transfected with Sp1 or Ctrl siRNA and treated with TNF (10 ng/ml) for 48 h (*n* = 5 to 7). (E) Quantitative PCR analysis of CPI-17 mRNA expression in rat aortic VSMCs transfected with Sp1 or Ctrl siRNA and treated with TNF for 48 h (*n* = 3 to 7). (F) Representative immunoblots of Sp1 and histone H3 protein expression in nuclear extracts from rat aortic VSMCs treated with TNF or saline. (G) Semiquantitative analysis of nuclear Sp1 immunoblots (*n* = 5). **, *P < *0.01; ***, *P < *0.001.

To directly test whether Sp1 is required for basal CPI-17 transcription and TNF-induced CPI-17 transcriptional suppression, we investigated whether pharmacological inhibition of Sp1 with mithramycin A affects basal and TNF-induced CPI-17 transcriptional suppression in rat aortic VSMCs treated or not treated with TNF (10 ng/ml, 48 h). Mithramycin A is an antibiotic with the ability to compete with Sp1 for binding to GC-rich DNA and has been shown to selectively inhibit Sp1 *in vitro* and *in vivo* (29). In the absence of mithramycin A (i.e., with a vehicle), a 51% reduction of CPI-17 promoter activity was observed in cells treated with TNF compared to that in control cells incubated with a vehicle (Fig. 5A). In contrast, in the presence of mithramycin A (50 nM, 48 h), a 23% reduction of CPI-17 promoter activity was found in cells treated with TNF compared to that in control cells incubated with the vehicle. In agreement with the role of Sp1 in basal CPI-17 transcription (18), mithramycin A inhibited basal CPI-17 promoter activity (Fig. 5A, solid bars) but had little effect on TNF-induced CPI-17 transcriptional suppression (Fig. 5A, open bars), suggesting that mithramycin A and TNF suppress CPI-17 transcription through inhibiting Sp1.

To more specifically define the role of Sp1 in TNF-induced CPI-17 transcriptional suppression, we investigated whether downregulation of Sp1 by small interfering RNAs (siRNAs) affects TNF-induced CPI-17 transcriptional suppression. As shown in Fig. 5B and C, transfection of Sp1-specific siRNA into rat aortic VSMCs (1 nM, 48 h) drastically diminished Sp1 protein expression relative to that in the control cells transfected with a scrambled siRNA. In agreement with the effect of inhibition of Sp1 by mithramycin A (Fig. 5A, solid bars), Sp1 siRNA also potently inhibited basal CPI-17 promoter activity (Fig. 5D, solid bars) and mRNA expression (Fig. 5E, solid bars) relative to those in cells transfected with control siRNA. In contrast to the minimal effect of inhibition of Sp1 by mithramycin A on TNF-induced CPI-17 promoter activity, Sp1 siRNA further diminished TNF-induced CPI-17 promoter activity (Fig. 5D, open bars) and mRNA expression (Fig. 5E, open bars) relative to those in cells transfected with control siRNA. Regardless of these differences, downregulation of Sp1 by siRNA, similar to inhibition of Sp1 by mithramycin A, completely abolished the effect of TNF on CPI-17 promoter activity (Fig. 5D, Sp1 siRNA versus Ctrl siRNA) and mRNA expression (Fig. 5E, Sp1 siRNA versus Ctrl siRNA).

To define the mechanism by which TNF inhibits the binding of Sp1 to the proximal GC boxes in the CPI-17 promoter, we explored the possibility that TNF decreases Sp1 nuclear accumulation and thereby attenuates the binding of Sp1 to the proximal GC boxes in CPI-17 promoter, thus suppressing CPI-17 transcription. Surprisingly, there was no difference in the levels of Sp1 nuclear accumulation between the cells treated and not treated with TNF (10 ng/ml, 48 h [Fig. 5F and G]), suggesting that additional transcription factors rather than Sp1 may directly respond to TNF to mediate CPI-17 transcription.

### KLF4 is upregulated by TNF, competes with Sp1 for the binding to the CPI-17 promoter, and represses CPI-17 transcription.

Genomatix MatInspector predicted several transcription factors putatively binding to the GC boxes in the CPI-17 promoter (data not shown). Among them, KLF4, in addition to Sp1, was particularly interesting, since KLF4 and Sp1 are in the same family of transcription factors that can bind to the GC boxes in the gene promoters with different sequence binding preferences and affinities and can function as activators or repressors depending on which promoter they bind and the coregulators with which they interact (28). Thus, we hypothesized that KLF4 might also be involved in TNF-induced CPI-17 transcriptional suppression through competing with Sp1 for the binding to the GC boxes in the CPI-17 promoter.

**FIG 6:**
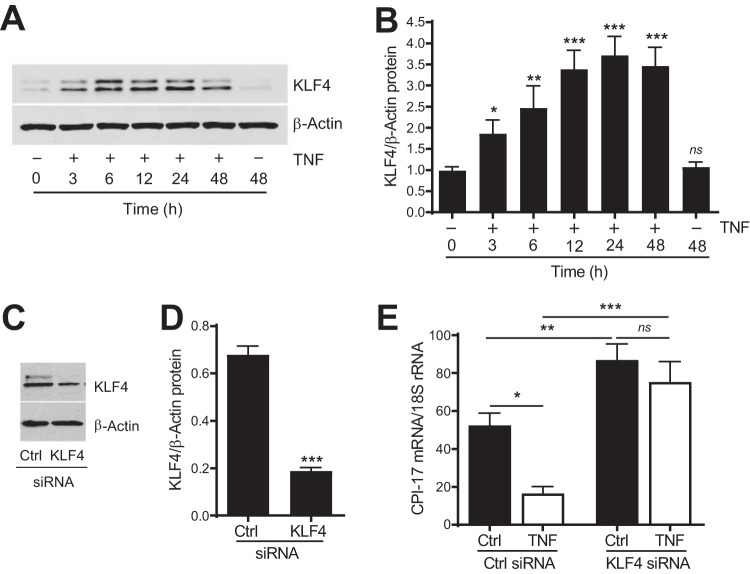
KLF4 is upregulated by TNF and is required for TNF-induced CPI-17 transcriptional suppression in VSMCs. (A) Representative immunoblots of KLF4 and β-actin protein expression in rat aortic VSMCs treated with TNF (10 ng/ml) or saline for the time indicated. (B) Semiquantitative analysis of KLF4 immunoblots (*n* = 6 to 9). (C) Representative immunoblots of KLF4 and β-actin protein expression in rat aortic VSMCs transfected with KLF4 siRNA (1 nM) or a scrambled siRNA (Ctrl) for 48 h. (D) Semiquantitative analysis of KLF4 immunoblots (*n* = 3). (E) Quantitative PCR analysis of CPI-17 mRNA expressions in rat aortic VSMCs transfected with KLF4 or Ctrl siRNA and then treated with TNF or saline (Ctrl) for 48 h (*n* = 3 to 7). *, *P < *0.05; **, *P < *0.01; ***, *P < *0.001; ns, not significant versus absence of TNF at time zero in panel B.

To test this hypothesis, we first determined whether KLF4 responds to TNF to be upregulated in cultured rat aortic VSMCs treated with TNF (10 ng/ml) for 3, 6, 12, 24, and 48 h. Representative blots (Fig. 6A) and quantitative data (Fig. 6B) demonstrated that KLF4 was significantly upregulated by TNF in VSMCs in a time-dependent manner relative to that in control cells (the cells at time zero). Interestingly, we found a double band of KLF4 protein (Fig. 6A) that has been previously described (30) and has been suggested to be related to KLF4 posttranslational modifications (31). It is worth pointing out that the observed upregulation of KLF4 was attributable to TNF but not the timing of cell culture since there was no difference in KLF4 expression between cells at 0 and 48 h in the absence of TNF (Fig. 6A and B). Moreover, TNF-induced KLF4 protein upregulation could be observed 3 h after TNF treatment (Fig. 6A and B), which preceded TNF-induced CPI-17 protein downregulation (Fig. 6A and B versus Fig. 2I and J, 12 h), suggesting that KLF4 may have a causal role in TNF-induced CPI-17 transcriptional suppression.

To directly test whether TNF-induced KLF4 protein upregulation causes TNF-induced CPI-17 transcriptional suppression, we investigated whether downregulation of KLF4 by siRNA affects TNF-induced CPI-17 mRNA suppression in cultured rat aortic VSMCs. As shown in Fig. 6C and D, transfection of KLF4 specific siRNA into cells (1 nM, 48 h) reduced KLF4 protein expression by 72% compared to that in the cells transfected with a scrambled control siRNA. Surprisingly, treatment of cells with the KLF4 siRNA increased rather than decreased CPI-17 mRNA expression relative to that in the cells treated with a scrambled control siRNA in the absence of TNF (Fig. 6E, solid bars). These results were sharply in contrast to the effect of downregulation of Sp1 by siRNA on CPI-17 mRNA expression in the absence of TNF (Fig. 6E versus Fig. 5E, solid bars), suggesting that KLF4 and Sp1 had opposite effects on CPI-17 transcription. Moreover, TNF-induced CPI-17 transcriptional suppression was abolished entirely in the cells treated with KLF4 siRNA relative to that in the cells treated with control siRNA (Fig. 6E, KLF4 siRNA versus Ctrl siRNA).

**FIG 7:**
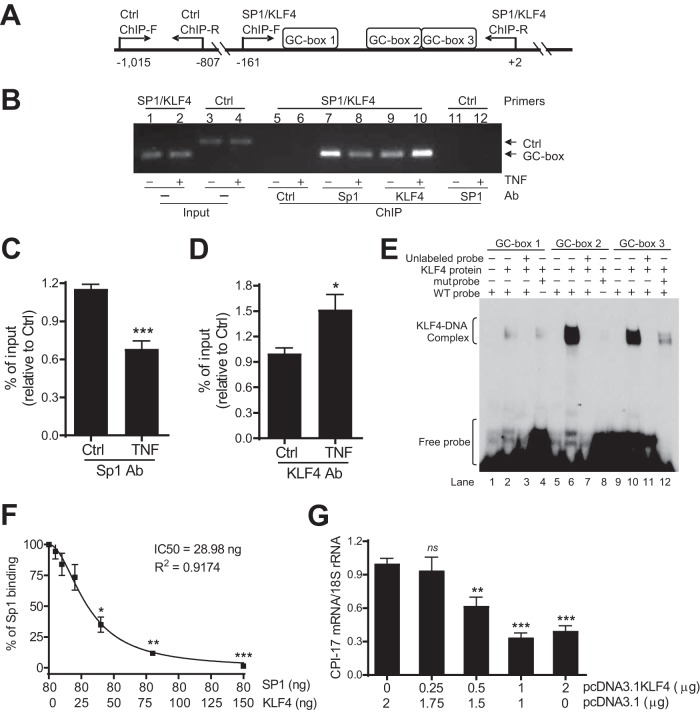
KLF4 competes with Sp1 for the binding of the same proximal GC boxes in the CPI-17 promoter in VSMCs. (A) Schematic diagram of ChIP-PCR for analysis of the binding Sp1 and KLF4 to proximal GC boxes in the CPI-17 promoter by Sp1/KLF4 ChIP-F (forward) and ChIP-R (reverse) primers relative to Ctrl ChIP-F and -R primers to amplify a region without GC boxes. (B) Representative ChIP-PCR showing a decreased Sp1 but increased KL4 binding to GC boxes in the CPI-17 promoter in cultured mouse aortic VSMCs treated with TNF (10 ng/ml, 48 h) relative to that in control cells. Ctrl ChIP-PCR ensured ChIP-PCR specificity. Ctrl Ab, a nonspecific control antibody to ensure Sp1 antibody specificity. (C and D) semiquantitative data of Sp1 and KLF4 ChIP-PCR (*n* = 6). (E) Representative EMSA showing the binding of purified KLF4 to WT GC boxes 2 and 3, but not mut GC box 2, or minimal binding to WT GC box 1 or mutant GC box 3 (*n* = 3). (F) Quantitative DPI-ELISA analysis of competition between KLF4 and Sp1 for the binding of GC box 2 in the CPI-17 promoter (*n* = 7). (G) Quantitative PCR analysis of CPI-17 mRNA expressions in rabbit aortic VSMCs transfected with various concentrations of pcDNA3.1 KLF4 as indicated (*n* = 10). *, *P < *0.05; **, *P < *0.01; ***, *P <* 0.001 versus Ctrl in panels C and D, Sp1 “80”/KLF4 “0” in panel F, and first solid bar in panel G.

To define the mechanism by which KLF4 participates in TNF-induced and Sp1-mediated CPI-17 transcriptional suppression, KLF4- and Sp1-bound chromatins were pulled down by antibodies specific for KLF4 and Sp1, respectively, in mouse aortic VSMCs treated or not treated with TNF (10 ng/ml, 48 h). The effect of TNF on the binding of KLF4 and Sp1 to the proximal GC boxes in the CPI-17 promoter was evaluated by ChIP-PCR (Fig. 7A). Consistent with the result shown in Fig. 4B and C, a decrease in Sp1 binding to the proximal GC boxes in the CPI-17 promoter was observed in cells treated with TNF compared to that in control cells without TNF treatment (Fig. 7B, lane 7 versus lane 8). In contrast, an increase in KLF4 binding to the same proximal GC boxes in the CPI-17 promoter was observed in cells treated with TNF compared to that in control cells without TNF treatment (Fig. 7B, lane 9 versus lane 10).

To ensure the specificity of the ChIP assay, two controls were included. First, a nonspecific control antibody ensured the specificity of Sp1 and KLF4 antibodies (Fig. 7B, lanes 5 and 6). Second, a pair of PCR primers to amplify a distal region of the CPI-17 promoter (bp −992 to −782) that does not contain GC boxes ensured the specificity of the binding of Sp1 or KLF4 to the proximal GC boxes in the CPI-17 promoter (Fig. 7B, lanes 11 and 12). Similar to the case with Fig. 4B and C, the ChIP PCR products were again normalized to the corresponding input and semiquantified by NIH ImageJ software. As shown in Fig. 4C and D, Sp1 binding to the proximal GC boxes in the CPI-17 promoter was reduced by 41%, whereas KLF4 binding to the same proximal GC boxes in the CPI-17 promoter was increased by 52%, respectively, in cells treated with TNF compared to that in the corresponding control cells without TNF treatment.

To investigate which proximal GC box in the CPI-17 promoter is bound by KLF4, a purified recombinant human KLF4 protein was incubated with ^32^P-labeled probes containing the proximal GC box 1, 2, or 3 in the CPI-17 promoter to form protein-DNA complexes. Purified KLF4 protein bound to all three proximal GC boxes in the CPI-17 promoter (Fig. 7E, lanes 2, 6, and 10), but the yields of the protein-DNA complexes were very different: many more protein-DNA complexes were found with probe 2 or 3 than with probe 1 (Fig. 7E, lane 2 versus lane 6 or 10). The observed KLF4-DNA complexes were specific, as little KLF4-DNA complex was observed in the absence of purified KLF4 protein (Fig. 7E, lanes 1, 5, and 9) or the presence of excessive unlabeled (cold) probes as competitors (Fig. 7E, lanes 3, 7, and 11). Interestingly, mutation of the proximal GC boxes in the CPI-17 promoter as described for Fig. 3C did not affect KLF4 binding to GC box 1 (Fig. 7E, lane 2 versus lane 4) but abolished KLF4 binding to GC box 2 (Fig. 7E, lane 6 versus lane 8) and GC box 3 (Fig. 7E, lane 10 versus lane 12).

To test whether upregulation of KLF4 by TNF can compete with Sp1 for binding to the same GC boxes in the CPI-17 promoter, we used purified KLF4 and Sp1 proteins and carried out an *in vitro* DNA-protein interaction enzyme-linked immunosorbent assay (DPI-ELISA) as described previously (32). A biotinylated DNA probe containing GC box 2 (2 pmol) of the CPI-17 promoter was first immobilized, then incubated with purified human Sp1 (80 ng), and then purified KLF4 (0 to 150 ng to mimic upregulation of KLF4 by TNF) to determine whether an increase in KLF4 can compete with Sp1 for the binding to the same GC box in the CPI-17 promoter. The yield of Sp1 that bound to the DNA probe was quantified by ELISA. As shown in Fig. 2G, KLF4 effectively competed with Sp1 for the binding to GC box 2 in the CPI-17 promoter in a concentration-dependent manner (Fig. 7F). The half-maximal inhibitory concentration (IC_50_) of KLF4 was 28.98 ng, indicating that KLF4 has a higher binding affinity for GC box 2 than Sp1.

To investigate whether upregulation of KLF4 by TNF caused CPI-17 transcriptional suppression, rabbit aortic VSMCs were transfected with various concentrations of human KLF4 cDNA (pcDNA3.1KLF4; 0.25 to 2 μg to mimic upregulation of KLF4 by TNF), and the effect of upregulation of KLF4 on CPI-17 mRNA expression was determined by real-time PCR. Rabbit aortic VSMCs were used because they have a relatively higher transfection efficiency than the rat or mouse aortic VSMCs. The same amount of plasmid DNA (pcDNA3.1KLF4 + pcDNA3.1 = 2 μg) was used for each sample to ensure similar transfection efficiencies. As shown in Fig. 7G, CPI-17 mRNA expression was inhibited by KLF4 in a concentration-dependent manner.

### Genetic deletion of TNF protects mice from LPS-induced smooth muscle CPI-17 downregulation, vasodilatory shock, and mortality.

**FIG 8:**
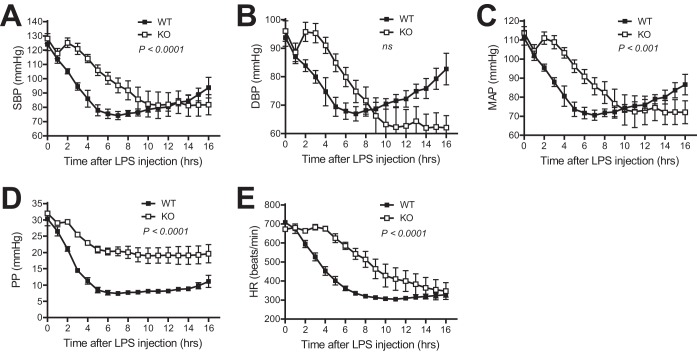
Genetic deletion of TNF protects mice from LPS-induced hypotension. Shown is quantitative analysis of SBP (A), DBP (B), MAP (C), PP (D), and HR (E) by telemetry in TNF knockout (KO; *n* = 4) and WT (*n* = 8) mice before and after LPS injection (10 mg/kg of body weight intravenously).

To investigate the physiological significance of the signaling pathway that we defined in cell culture and a test tube, we determined whether blockade of TNF/KLF4/SP1/CPI-17 signaling protects mice from LPS-induced hypotension. We first investigated whether genetic deletion of TNF protects mice from LPS-induced hypotension. TNF knockout (TNF-KO) and WT control mice were injected intravenously with LPS (10 mg/kg), and systolic BP (SBP), diastolic BP (DBP), mean arterial pressure (MAP), pulse pressure (PP), and heart rate (HR) were measured by telemetry before and after LPS injection. Before LPS injection (0 h in Fig. 8), there was no difference in SBP, DBP, MAP, PP, and HR (Fig. 8), suggesting that genetic deletion of TNF has minimal effect on basal BP and HR. After LPS injection, both TNF-KO and WT mice exhibited a time-dependent decrease in SBP, DBP, MAP, PP, and HR, but the rate of decrease in TNF-KO mice was delayed compared with that in WT mice (Fig. 8), indicating that genetic deletion of TNF protects mice, at least in part, from LPS-induced hypotension. However, it was noted that the protection by genetic deletion of TNF was observed only in the early times (up to 8 to 10 h after LPS injection). Surprisingly, an increase rather than a further decrease in LPS-induced BP change was observed 8 h after LPS injection in WT mice but not in TNF-KO mice (Fig. 8A to C).

**FIG 9:**
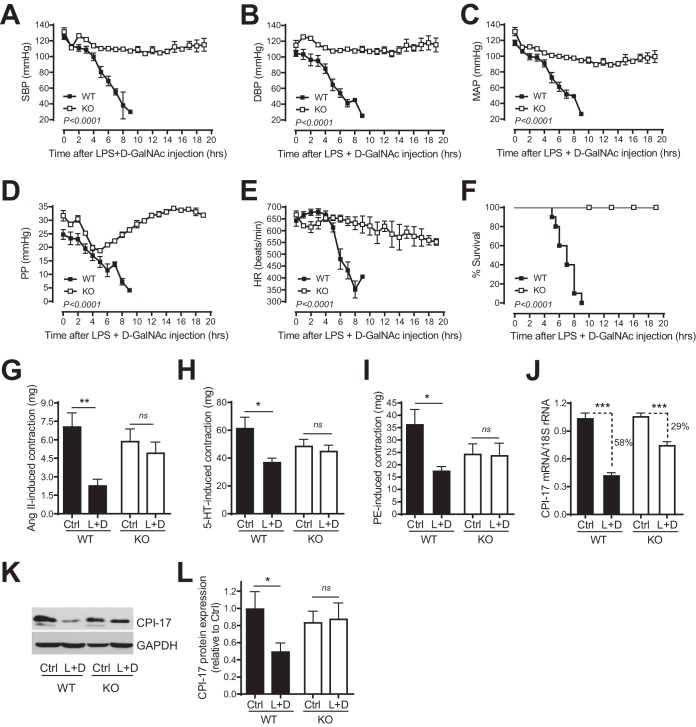
Genetic deletion of TNF protects mice from smooth muscle CPI-17 downregulation, vascular hypocontractility, hypotension, and mortality induced by LPS plus d-GalNAc. (A to E) Quantitative analysis of SBP (A), DBP (B), MAP (C), PP (D), and HR (E) by telemetry in TNF knockout (*n* = 4) and WT (*n* = 8) mice before and after LPS (0.5 mg/kg of body weight intraperitoneally [i.p.]) plus d-GalNAc (1 g/kg of body weight i.p.) injection. (F) Quantitative analysis of survival of TNF knockout (*n* = 5) and WT (*n* = 10) mice injected with LPS plus d-GalNAc. (G, H, and I) Quantitative analysis of vascular smooth muscle contractile responses to Ang II (100 nM) (G), 5-HT (10 μM) (H), and PE (10 μM) (I) by isometric tension measurement in mesenteric arteries from TNF-KO and WT mice injected with LPS plus d-GalNAc (L+D) or saline (Ctrl) (*n* = 4 or 5). (J) Quantitative PCR analysis of CPI-17 mRNA expression in mesenteric arteries from TNF-KO and WT mice 6 h after injection with LPS plus d-GalNAc or saline (*n* = 5). (K and L) Representative immunoblots (K) and semiquantitative analysis (L) of CPI-17 and β-actin protein expression in mesenteric arteries from TNF-KO and WT mice injected with LPS plus d-GalNAc or saline (*n* = 5). *, *P < *0.05; **, *P < *0.01; ***, *P < *0.001.

We were unable to define the role of TNF in LPS-induced mortality since none of the mice died during the 16-h period after LPS injection. To address this critical issue, TNF-KO and WT control mice were injected intraperitoneally with a low dose of LPS (0.5 mg/kg) plus d-galactosamine (d-GalNAc; 100 mg/kg). d-GalNAc is an LPS-sensitizing reagent. In the presence of d-GalNAc, a lower dose of LPS can potently and rapidly induce septic endotoxin shock and animal death that are entirely dependent upon TNF (22, 33). As shown in Fig. 9A to E, SBP, DBP, MAP, PP, and HR rapidly dropped down to the lethal level 9 h after injection of LPS plus GalNac in the WT mice. In striking contrast, SBP, DBP, MBP, PP, and HR in TNF-KO mice were mostly resistant to the effect of LPS plus d-GalNAc (Fig. 9A to E, KO versus WT). Moreover, all WT mice died 9 h after injection of LPS plus d-GalNAc, but all TNF-KO mice survived after injection of LPS plus d-GalNAc (Fig. 9F).

To investigate whether such striking protection of mice from hypotension and mortality by genetic deletion of TNF is associated with the protection from vascular hypocontractility, we determined smooth muscle contractile responses to Ang II, 5-HT, and PE in mesenteric arteries from TNF-KO and WT control mice injected with LPS plus d-GalNAc. As expected, smooth muscle contractile responses to angiotensin II (Ang II; 100 nM), serotonin (5-HT; 10 μM), and PE (10 μM) were reduced in mesenteric arteries from mice injected with LPS plus d-GalNAc compared to that in WT control mice (Fig. 9G to I). In contrast, smooth muscle contractile responses to Ang II, 5-HT, and PE in mesenteric arteries were preserved in the TNF-KO mice injected with LPS plus d-GalNAc (Fig. 9G to I).

To further investigate whether the protection from vascular hypocontractility by genetic deletion of TNF is associated with the protection from CPI-17 downregulation, we determined CPI-17 mRNA and protein expression in mesenteric arteries from TNF-KO and WT control mice injected with LPS plus d-GalNAc. As expected, CPI-17 mRNA and protein were significantly downregulated in mesenteric arteries from WT mice injected with LPS plus d-GalNAc compared to that in WT control mice (Fig. 9J to L). In contrast, CPI-17 mRNA and protein downregulations induced by LPS plus d-GalNAc were attenuated or abolished in mesenteric arteries from TNF-KO mice compared to those in WT control mice (Fig. 9J to L).

### Pharmacological inhibition of HDACs blocks KLF4-induced CPI-17 transcriptional suppression and protects mice from hypotension and mortality induced by LPS plus d-GalNAc.

**FIG 10:**
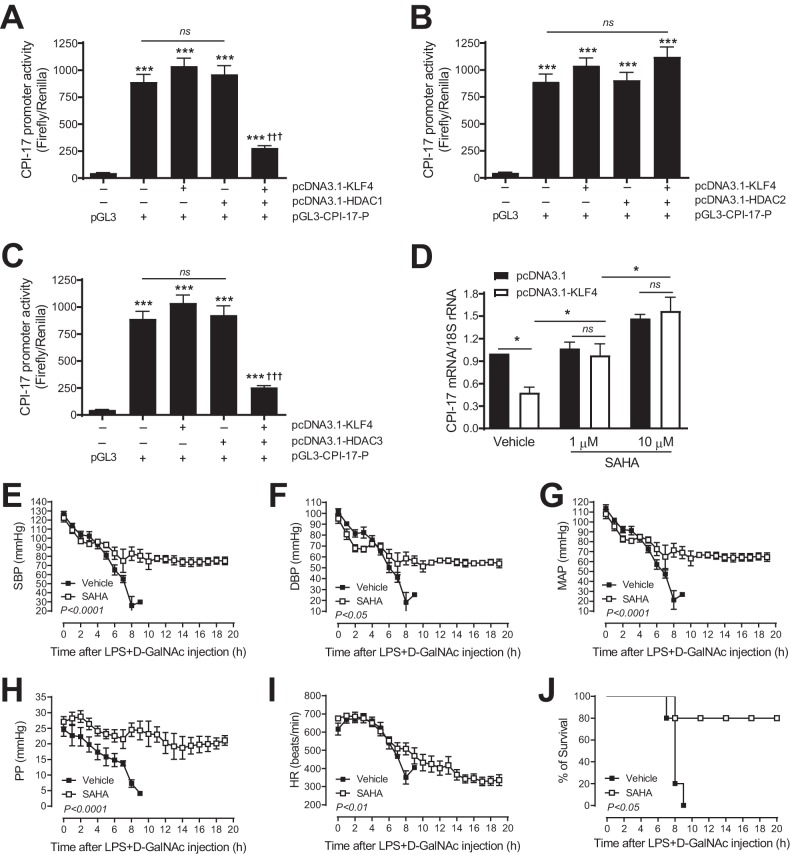
Pharmacological inhibition of HDACs blocks KLF4-induced CPI-17 transcriptional suppression and protects mice from hypotension and mortality induced by LPS plus d-GalNAc. (A to C) Analysis of CPI-17 promoter activity in rabbit aortic VSMCs cotransfected with pGL3-CPI-17 promoter-luciferase construct (bp −392) and pCDNA3.1-KLF4 (25 ng) with pCDNA3.1-HDAC1 (25 ng) (A), pcDNA3.1-HDAC2 (25 ng) (B), and pCDNA3.1-HDAC3 (10 ng) (C) vectors for 48 h (*n* = 3). (D) Quantitative PCR analysis of CPI-17 mRNA expression in rabbit aortic VSMCs transfected with pCDNA3.1-KLF4 (2 μg) and then treated with HDAC inhibitor SAHA for 48 h (*n* = 4). (E to I) Quantitative analysis of SBP (E), DBP (F), MAP (G), PP (H), and HR (I) by telemetry in C57BL/6 mice injected with LPS plus d-GalNAc and treated with SAHA (50 mg/kg of body weight) or a vehicle (DMSO) (*n* = 5). (J) Quantitative analysis of survival of C57BL/6 mice injected with LPS plus d-GalNAc and treated with SAHA and a vehicle (*n* =5). ***, *P *< 0.001 versus pGL3 in panels A, B, and C; †††, *P *< 0.001 versus pcDNA3.1-KLF4 or pcDNA3.1-HDAC1 alone in panel A, pcDNA3.1-KLF4 or pcDNA3.1-HDAC2 alone in panel B, and pcDNA3.1-KLF4 or pcDNA3.1-HDAC3 alone in panel C.

It has been shown that KLF4 interacts with HDAC2 to mediate PDGF-induced SM22α transcriptional silencing during VSMC phenotypic switching (34). It has also been shown that cotransfection of KLF4 with HDAC3 synergistically represses cycling B1 transcription in human colon cancer cells (35). Thus, we wondered whether HDACs are involved in KLF4-induced CPI-17 transcriptional suppression. We first cotransfected rabbit aortic VSMCs with various concentrations of KLF4, HDACs, and CPI-17 promoter constructs and determined the optimal concentrations of KLF4 and HDACs that show synergistic effects: transfection of either KLF4 or HDACs alone did not, but cotransfection of KLF4 with HDACs did, repress CPI-17 transcription (data not shown). As shown in Fig. 10A to C, cotransfection of cells with KLF4 and HDAC1 or HDAC3, but not HDAC2, resulted in synergistic repression of CPI-17 transcription, indicating that KLF4 represses CPI-17 transcription via HDACs.

To test whether KLF4 represses CPI-17 transcription through HDACs, rabbit aortic VSMCs were first transfected with a high concentration of KLF4 (2 μg) that was sufficient to inhibit CPI-17 transcription (Fig. 7G). Cells were then treated with suberoylanilide hydroxamic acid (SAHA; 1 and 10 μM) or a vehicle (dimethyl sulfoxide [DMSO]) for 48 h. SAHA (also known as vorinostat) is a potent inducer of a broad range of HDACs, including HDAC1, HDAC2, and HDAC3 (36). The effect of SAHA on basal and KLF4-induced CPI-17 mRNA expression was determined by real-time PCR. We hypothesized that if KLF4 represses CPI-17 transcription through HDACs, inhibition of HDACs with SAHA would be expected to reverse KLF4-induced CPI-17 transcriptional suppression. The results supported our hypothesis. As shown in Fig. 10D, in the absence of SAHA (i.e., with a vehicle), CPI-17 mRNA expression was inhibited in cells transfected with pcDNA3.1-KLF4 vector compared to that in control cells transfected with pcDNA3.1 backbone vector. In contrast, in the presence of 1 or 10 μM SAHA, there were no differences in CPI-17 mRNA expression between cells transfected with pcDNA3.1 and pcDNA3.1-KLF4 vectors (Fig. 10D, open bars versus solid bars).

To investigate the physiological relevance of our *in vitro* finding that KLF4 represses CPI-17 transcription through HDACs, C57BL/6 mice were injected intraperitoneally with SAHA (50 mg/kg) before being injected intraperitoneally with LPS (0.5 mg/kg) plus d-GalNAc (100 mg/kg). The effect of SAHA on hypotension induced by LPS plus d-GalNAc was determined by telemetry. As shown in Fig. 10E to I, pharmacological inhibition of HDACs with SAHA protected mice, at least in part, from a sudden drop in SBP, DBP, MAP, PP, and HR induced by LPS and d-GalNAc. Consistent with these data, mortality induced by LPS and d-GalNAc was also significantly reduced in mice treated with SAHA compared to that in the control mice treated with a vehicle (DMSO).

## DISCUSSION

**FIG 11:**
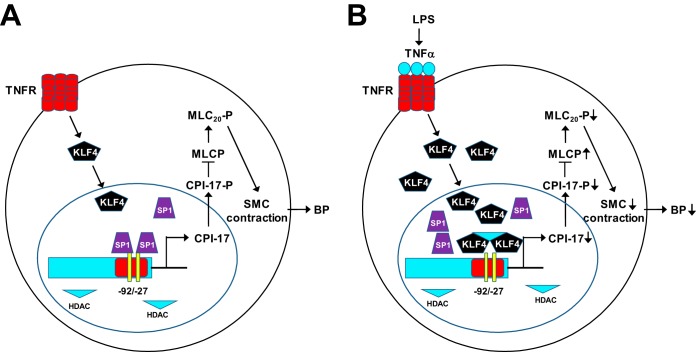
A proposed potential mechanism for TNF-induced CPI-17 transcriptional suppression in VSMCs in a mouse LPS model of sepsis. (A) Under physiological conditions, Sp1 binds to proximal GC boxes 2 and 3 in the CPI-17 promoter, activates CPI-17 transcription, and promotes high CPI-17 expression in arterial smooth muscle, thus maintaining normal vascular tone and BP homeostasis. (B) Under a pathological condition (e.g., endotoxemia induced by LPS), KLF4 is markedly upregulated by TNF, competes with Sp1 for binding to the same GC boxes in the CPI-17 promoter, represses CPI-17 transcription through HDAC1 and HDAC2, and reduces CPI-17 expression in arterial smooth muscle, thus leading to vasodilatory shock and hypotension.

The significant new findings from current studies are that we identified a key *cis* TNF response element (GC boxes) in the CPI-17 promoter critical for TNF-induced and KLF4- and Sp1-mediated CPI-17 transcriptional suppression in VSMCs. Mechanistically, we demonstrated that KLF4 is upregulated by TNF, competes with Sp1 for the binding of the same GC boxes in the CPI-17 promoter, and represses CPI-17 transcription via HDACs (Fig. 11). The significance of these findings is highlighted by the facts that currently there is no effective medication for the treatment of vasodilatory shock in patients with sepsis, and inhibition of the TNF/KLF4/Sp1/HDAC/CPI-17 signaling by genetic deletion of TNF or pharmacological inhibition of HDACs protects mice from LPS-induced hypotension, vascular hypocontractility, and mortality.

Vasodilatory shock has been extensively investigated due to its clinical implications. Among several proposed potential mechanisms for vasodilatory shock, excessive production of NO by iNOS due to inflammation in sepsis has been suggested to be a major and central player in vasodilatory shock (2, 4, 5). It is generally agreed that NO generated by iNOS activates soluble guanylyl cyclase (sGC), results in elevation of intracellular cyclic GMP (cGMP) levels and activation of protein kinase G (PKG), and thus leads to smooth muscle relaxation, vasodilatory shock, and hypotension (2, 4, 5). Therefore, there have been the extensive animal and human studies that aimed to inhibit iNOS/NO/sGC/cGMP/PKG signaling to restore the smooth muscle contractile response (2, 4, 5). Recently, it was reported that vasodilator shock and hypotension were suppressed in oxidation-resistant PKG Iα mice in sepsis mouse models of LPS and cecal ligation and puncture (17, 37). However, it should be pointed out that use of NOS inhibitors in animals and patients with sepsis was only partially successful in improving vasodilatory shock and hypotension and did not improve survival (2, 4, 5). The primary challenge in using NOS inhibitors to treat vasodilator shock is that NOS inhibitors not only inhibited iNOS, which is beneficial, but also inhibited endothelial NOS (eNOS), which may result in detrimental NO deficiency (2, 4, 5).

In contrast to the extensive research on the role of NO in vasodilatory shock, there have been few studies focusing on the potential role of the intrinsic VSMC contractile defect in vasodilatory shock. da Silva-Santos et al. reported that in mesenteric arteries from endotoxemic rats, phosphorylated MYPT1, an index of ROCK activation, was significantly reduced, although RhoA, ROCK-I, ROCK-II, and MYPT1 were increased, suggesting that the inhibition of the RhoA/ROCK pathway may be responsible for vasodilatory shock (19). Reho et al. recently reported that smooth muscle myosin and actin, MLCK, MYPT1, and CPI-17 were all diminished in mesenteric arteries from endotoxemic mice (17), suggesting further that the defect in smooth muscle contractile machinery, including CPI-17, may play a role in vasodilatory shock. In agreement with this finding, the current study demonstrated a striking selective downregulation of CPI-17 among several smooth muscle contractile machinery proteins by LPS in mesenteric arteries, suggesting that downregulation of CPI-17 may be specifically involved in vasodilatory shock.

The mechanism by which LPS downregulates smooth muscle CPI-17 is unknown. Several lines of evidence from the current study demonstrated that TNF links LPS and smooth muscle CPI-17 downregulation. First, TNF upregulation was spatially associated with CPI-17 downregulation in mesenteric arteries from mice injected with LPS. Second, injection of mice with TNF mimicked LPS to downregulate CPI-17 in mesenteric arteries. Third, incubation of isolated aortas and cultured VSMCs with TNF was sufficient to induce CPI-17 downregulation. Finally, genetic deletion of TNF abolished CPI-17 downregulation induced by LPS plus d-GalNAc in mesenteric arteries. However, it was noted that there are inconsistent reports regarding the effect of TNF on smooth muscle CPI-17 protein expression and vascular function. Ohama et al. reported that CPI-17 was downregulated by TNF in rat ileum smooth muscle tissues, in concordance with hypocontractility (11). In contrast, Morin et al. reported that CPI-17 was upregulated by TNF in human bronchus smooth muscle tissues, in concordance with hypercontractility (38). Kim et al. reported that CPI-17 was upregulated by TNF in rat aortic VSMCs (18). Yang et al. reported that TNF increased cerebral artery myogenic tone in a heart failure mouse model (39). These conflicting results may be related to different cell types or experimental conditions. Thus, the effects of TNF on CPI-17 and vascular function perhaps are dependent upon the context.

The mechanism by which TNF downregulates smooth muscle CPI-17 is also unknown. Kim et al. first reported that Sp1 bound to the GC boxes in the proximal CPI-17 promoter and activated CPI-17 transcription in VSMCs (18). By 5′ promoter deletion analysis and site-directed mutagenesis, the current studies independently identified the proximal GC boxes in the CPI-17 promoter as a key *cis* TNF response element critical for NF-induced CPI-17 transcriptional suppression. However, it should be pointed out that since deletion/mutation of the CPI-17 promoter caused loss of both basal and TNF-induced CPI-17 transcriptional suppression, the effect of deletion/mutation on basal activity may confound the effect on TNF-induced CPI-17 transcriptional suppression. Regardless of this confounding effect, one of the most important findings from the current study is that we further demonstrated that KLF4, but not Sp1, was upregulated by TNF in VSMCs. Mechanistically, we showed that KLF4 competes with Sp1 for binding to the same proximal GC boxes in the CPI-17 promoter in a concentration-dependent manner. By pharmacological inhibition and siRNA downregulation, we demonstrated that both Sp1 and KLF4 were required for TNF-induced CPI-17 transcriptional suppression, but they exhibited opposite effects: Sp1 activated CPI-17 transcription, whereas KLF4 repressed CPI-17 transcription.

How is it that Sp1 and KLF4 bind to the same GC boxes but exhibit opposite effects on CPI-17 transcription? Sp1 has been recognized for its ability to recruit TATA-binding protein (e.g., RNA polymerase II) and fix the transcriptional start site at TATA-less promoters (28). KLF4, on the other hand, has been recognized for its ability to recruit HDACs, allowing the histones to wrap the DNA more tightly and thus repress smooth muscle marker gene expression (40). Histone acetylation and deacetylation are a reversible and dynamic histone modification process, controlled, respectively, by the antagonistic actions of two large families of enzymes: the histone acetyltransferases (HATs) and HDACs (41). Salmon et al. reported that cooperative binding of KLF4 and HDAC2 to a G/C repressor element in the SM22α promoter mediated transcriptional silencing during SMC phenotypic switching *in vivo* (34). Interestingly, the current study demonstrated that KLF4 had a synergistic effect on CPI-17 transcription with HDAC1 or HDAC3, but not HDAC2, in VSMCs, indicating that the interaction between KLF4 and HDACs is promoter specific.

The current study has several limitations. First, this study has not established that downregulation of CPI-17 is the mechanism by which LPS or TNF mediates vasodilatory shock, hypotension, and mortality. Further studies on CPI-17 knockout mice injected with LPS or TNF may help to address this critical issue. Second, whether the minimal CPI-17 promoter tested *in vitro* mediates TNF-induced CPI-17 mRNA downregulation occurs *in vivo* remains to be determined. There are likely enhancer elements elsewhere within or beyond the gene that confer tight transcriptional control of CPI-17. Sequence analyses by Dippold and Fisher, who identified conserved putative enhancer sequences in introns 2 and 3 of the CPI-17 gene, support this possibility (42). Third, two different mouse LPS models of sepsis were used in the current study. Compared with the high-dose LPS model, the low-dose LPS plus d-GalNAc model rapidly induced animal death that was entirely dependent upon TNF (22, 33), thus allowing a better evaluation of the effect of TNF on LPS-induced mortality. However, it should be pointed out that the mouse LPS model of sepsis was considered an endotoxemia model rather than sepsis model (43), and thus, the results of the current study may have limitations in their applicability. Fourth, the use of three chemicals (LPS, d-GalNAc, and SAHA) may compound the experimental results because of possible chemical interactions. Finally, the current study is not sufficient to exclude a role for NF-κB in TNF-induced transcriptional suppression since the current study investigated only one of the putative NF-κB binding sites in the CPI-17 promoter in TNF-induced transcriptional suppression.

## MATERIALS AND METHODS

### Animals.

TNF knockout mice (TNF-KO) and wild-type (WT) C57BL/6J mice were purchased from the Jackson Laboratory. All mice were housed at an animal care facility at the University of Kentucky. Only male mice at 12 to 14 weeks of age were used. All animal procedures were approved by the Institutional Animal Care and Use Committee of the University of Kentucky.

### Administration of LPS, TNF, and other compounds to animals.

As explicitly described in Results, LPS, TNF, and other compounds were administered to mice by intravenous or intraperitoneal injection as follows. For high-dose LPS, mice were injected intravenously with LPS (Escherichia coli O111:B4; Sigma-Aldrich; 10 mg/kg of body weight in 100 μl of saline for 6 or 16 h). For TNF injection, mice were injected intravenously with TNF (recombinant mouse TNF; BioLegend; 6 μg in 100 μl of saline per mouse for 6 h). For low-dose LPS plus d-galactosamine (d-GalNAc), mice were injected intraperitoneally with LPS (0.5 mg/kg of body weight in 100 μl of saline for 20 h) plus d-GalNAc (Sigma-Aldrich; 1 g/kg of body weight in 100 μl of saline for 20 h). For suberoylanilide hydroxamic acid (SAHA), mice were injected intraperitoneally with SAHA (Sigma-Aldrich; 50 mg/kg of body weight in 100 μl of dimethyl sulfoxide [DMSO] for 20 h).

### Telemetric measurement of blood pressure.

The procedures for telemetric measurement of blood pressure were conducted as previously described (9, 25, 44). Briefly, mice were instrumented in the left common carotid artery with a telemetry probe (TA11PA-C10; Data Sciences International). After 10 days of recovery, systolic BP (SBP), diastolic BP (DBP), mean arterial pressure (MAP), pulse pressure (PP), and heart rate (HR) were collected before, during, and after LPS injection.

### Semiquantitative analysis of protein expression and phosphorylation.

The procedures for semiquantitative analysis of protein expression and phosphorylation by Western blotting were performed as previously described (9, 13, 14, 23, 25, 45, 46). The antibodies used in immunoblots are described in Table S1 in the supplemental material.

### IHC.

The procedures for using immunohistochemistry (IHC) to determine *in situ* protein expression in paraffin-embedded mouse mesenteric arteries were performed as previously described (9, 25, 45, 46). The antibodies used for IHC are described in Table S1.

### Quantitative analysis of mRNA expression.

The procedures for RNA extraction, cDNA synthesis, and real-time PCR were performed as previously described (13, 23, 25, 26, 45–48). The PCR primers for amplifying rat CPI-17 and mouse platelet-derived growth factor subunit B (PDGF-B) are described in Table S2. The PCR primers for amplifying mouse CPI-17, ROCK2, TNF, and 18S RNA were previously described (13, 23, 25, 26, 45–48).

### Aortic organ culture.

Aortas were isolated from C57BL/6 mice. After dissection to remove fat and adventitial tissues, aortas were cut into 3-mm segments. Endothelial cells were mechanically removed. Aortic segments were incubated with purified recombinant mouse TNF (10 ng/ml; R&D Systems) for 48 h in Krebs-Ringer bicarbonate buffer containing penicillin and streptomycin. The incubation buffer containing TNF was replaced every 12 h to ensure the efficacy of TNF.

### Primary cell culture.

The procedures for isolating and culturing aortic VSMCs from mice, rats, and rabbits were conducted as previously described (13, 14, 23, 26, 47). Cells were starved in serum-free medium overnight and then treated with TNF (10 ng/ml) in serum-free medium for the times specified in Results. The incubation buffer containing TNF was replaced every 12 h to ensure the efficacy of TNF.

### Cloning CPI-17 promoters.

A bacterial artificial chromosome (BAC) clone containing the mouse CPI-17 promoter was purchased from Invitrogen and was used as a PCR template to amplify the bp −792 promoter. After sequencing (ACGT, Inc.), the bp −792 promoter was used as a PCR template to amplify the bp −592, −392, −203, and −92 promoters. The PCR primers to amplify all of these promoters are described in Table S2. The bp −27 promoter was synthesized by Integrated DNA Technologies (IDT). All CPI-17 promoters were subcloned into a pGL3-basic vector (Promega) as previously described (25, 26, 47).

### Site-directed mutagenesis.

A QuikChange II site-directed mutagenesis kit (Agilent Technologies) was used to generate mutations in the CPI-17 promoter. The PCR primers used for generating mutations of GC boxes 1, 2, and 3 and NF-κB in the CPI-17 promoters are described in Table S2. All mutations were verified by DNA sequencing.

### CPI-17 promoter assay.

Rat aortic VSMCs were cotransfected with pGL3-CPI-17 firefly luciferase vectors and a *Renilla* luciferase control vector (pRL-Null; Promega) using Lipofectamine-Plus reagent (Life Technologies). After TNF treatment (10 ng/ml, 48 h), CPI-17 promoter activity was assayed by a modified dual-luciferase enzyme assay as described previously (25, 26, 47).

### siRNA downregulation.

Rat aortic VSMCs were transfected with 1 nM rat Sp1- or KLF4-specific siRNA duplex (Santa Cruz Biotech) or nonspecific siRNA duplex (Qiagen) using INTERFERin reagent (Polyplus-transfection Inc.) for 48 h. The specificity and efficiency of siRNA downregulation of Sp1 and KLF4 were determined by Western blotting.

### EMSA.

Electrophoretic mobility shift assay (EMSA) was conducted as previously described (26). Briefly, nuclear extracts (2 μg) from rat aortic VSMCs treated with TNF or a purified recombinant human KLF4 protein (50 ng; Sigma-Aldrich) were incubated for 30 min on ice with ^32^P-labeled double-stranded DNA probes corresponding to WT or mutant GC boxes 1, 2, and 3 in the CPI-17 promoter. To ensure the specificity of the assay, excess unlabeled probes (100:1) were added to the reaction mixture. To determine whether Sp1 binds to the GC boxes, 0.5 or 1 μg of anti-Sp1 antibody (Santa Cruz Biotech) was included in the reaction mixture. The WT and mutant probes used for EMSA are described in Table S2. All probes were synthesized by IDT and labeled with [γ-^32^P]ATP (3,000 Ci/mmol; Perkin Elmer Life Sciences) by T4 kinase (New England BioLabs). Reaction mixtures were analyzed on 6% nondenaturing polyacrylamide gels, followed by autoradiography.

### ChIP assay.

The chromatin immunoprecipitation (ChIP) assay was conducted as previously described (25). Mouse primary aortic VSMCs were starved in serum-free medium and then treated with TNF (10 ng/ml, 48 h). Cells were fixed with 1% formaldehyde for 15 min to preserve the protein-DNA interactions. After quenching by glycine, cells were lysed in cold cell lysis buffer containing protease inhibitors. Five of the 10-cm dishes of cells were pooled for one sample. After cells were lysed, cell nuclei were collected by centrifugation and resuspended in 0.5 ml of nucleus lysis buffer for ultrasonication (Branson Digital Sonifier 450; microtip; 10 times for 10 s with an interval of 10 s and 36% power). After centrifugation, the supernatant containing fragmented chromatins was diluted 10-fold, and 50 μl of diluted chromatins was collected as an input. One milliliter of diluted chromatins was precleared by incubation with salmon sperm DNA (Invitrogen) and protein A/G-agarose beads (Santa Cruz Biotech) and then subjected to immunoprecipitation with the Sp1 or KLF4 antibodies (2 μg; Santa Cruz Biotech) or nonspecific rabbit IgG (2 μg; Vector Laboratories) overnight at 4°C. After washing and centrifugation, immune complexes were eluted and then were heated at 65°C for 4 h to reverse protein-DNA cross-links. Purified DNA was used a ChIP-PCR template to amplify GC boxes of the CPI-17 promoter. The ChIP-PCR primers are described in Table S2.

### DPI-ELISA.

A DNA-protein interaction enzyme-linked immunosorbent assay (DPI-ELISA) was conducted as previously described (32). Briefly, a biotinylated double-stranded (ds-bio) DNA oligonucleotide containing the proximal GC box 2 in the mouse CPI-17 promoter (Table S2) was purchased from IDT. Two picomoles of ds-bio oligonucleotides was immobilized on streptavidin-coated 96-well plates (R&D Systems). After blocking and washing, the coated microtiter plates were incubated first with a purified recombinant human Sp1 (80 ng; OriGene) and then with purified recombinant human KLF4 (0 to 150 ng; MyBioSource) for 2 h. After washing, the binding of Sp1 to ds-bio oligonucleotides was detected with a rabbit anti-Sp1 antibody (Cell Signaling) followed by an anti-rabbit antibody conjugated with horseradish peroxidase.

### Isometric tension measurement.

The procedures for mesenteric artery isometric contraction measurement were initially reported by Horiuti (49) and conducted as previously described (13, 25, 48, 50, 51). Briefly, mesenteric arteries were isolated from TNF-KO and control C57BL6 mice 6 or 16 h after LPS injection. After removal of fat and adventitial tissues, 1 second-order branch of mesenteric arteries was cut into a small spiral strip (about 3 mm in length and 350 μm in width). Endothelial cells were denuded by a gentle scrape with a razor blade, and the successful denudation was verified by the loss of maximal-dose acetylcholine (1 mmol/liter)-induced relaxation. The two ends of muscle strips were tied to two tungsten wire hooks with monofilament thread and stretched to 1.3 times their resting lengths. One of the tungsten wires was attached to a force transducer (AE, 801; SensoNor, Horten, Norway) (49). The force transducer was calibrated with a standard weight set (5 mg, 10 mg, and 20 mg) before it was used for isometric tension measurement. Thus, the unit of isometric tension measurement was milligrams, but it could be expressed as Newton or percentage of maximal contraction induced by high potassium as previously described (13, 25, 48, 50, 51). Isometric tension was measured at 24°C in a well on a “bubble” plate. The solution was changed completely by immersing the muscle trip into an adjacent bubble by sliding the bubble plate. The muscle trips were stimulated with 154 mmol/liter of high-potassium solution 3 or 4 times until a stable response was obtained. Then, PE (10 μM), serotonin (5-HT; 10 μM), and angiotensin II (Ang II; 100 nM) were added to induce smooth muscle contractions. The total time of the *in vitro* experiments from vessel excision was usually 6 h.

### Statistics.

All data were expressed as means ± standard errors of the means (SEM). For a comparison of 1 parameter between 2 samples, statistical analysis was performed using 2-tailed, unpaired Student’s *t* test. For a comparison of 1 parameter among multiple samples, statistical analysis was performed using 1-way analysis of variance (ANOVA) with a Newman-Keuls posttest. For comparison of multiple parameters, statistical analysis was performed using 2-way ANOVA with repeated measures and Bonferroni’s posttest. A *P* value of <0.05 was considered significant. A *P* value of ≥0.05 was considered nonsignificant (ns).

## Supplementary Material

Supplemental file 1
