# Supplementary material for: Transcriptional Suppression of CPI-17 Gene Expression in Vascular Smooth Muscle Cells by Tumor Necrosis Factor, Krüppel-Like Factor 4, and Sp1 Is Associated with Lipopolysaccharide-Induced Vascular Hypocontractility, Hypotension, and Mortality
Source: Mol Cell Biol. 2019 May 14;39(11):e00070-19. doi: 10.1128/MCB.00070-19 (PMC6517596; doi:10.1128/MCB.00070-19)
Supplement: Supplemental file 1 [file MCB.00070-19-s0001.pdf]

Table 1. Antibodies used in the current study.

Antibody	Company & Catalog number	Species	Application	Dilution used	Protein Size	Cited References
β-Actin	Cell Signaling 4967	Human, rat mouse	Western Blot	1:2000	42 kDa	*J Biol Chem.* 2010 285(12):8628-38
CPI-17	Gene Tex GTX61218	Human, rat mouse	Western Blot	1:2000	17 kDa	None.
CPI-17	Genemed Synthesis Custom Ab	Human, rat mouse	Western blot IHC	1:2000 1:200	17 kDa	*Am J Physiol Cell Physiol.* 2005 289(2):C352-60
CPI-17	EMD, Millipore 07-344	Human, rat mouse	Western Blot	0.2 μg/ml 2.5 μg/ml	17 kDa	*Am J Physiol Heart Circ Physiol.* 2013 305(1):H104-13
Phospho CPI-17	Genemed Synthesis Custom Ab	Human, rat mouse	Western Blot	1:2000	17 kDa	*Am J Physiol Cell Physiol.* 2005 289(2):C352-60
KLF4	Cell Signaling 4038	Human, rat mouse	Western Blot	1:2000	65 kDa	*J Biol Chem.* 2012 287(14):10799-811
KLF4	Santa Cruz Biotech sc-20691	Human, rat mouse	ChIP	2 μg	65 kDa	*J Biol Chem.* 2012 287(14):10799-811
MLC_{20}	Cell Signaling 3672	Human, rat mouse	Western Blot	1:2000	18 kDa	*J Clin Invest.* 2015 125(1):324-36
Phospho MLC_{20}	Cell Signaling 3675	Human, rat mouse	Western Blot	1:2000	18 kDa	*J Clin Invest.* 2015 125(1):324-36
PKCα	BD Biosciences 610107	Human, rat mouse	Western Blot	1:2000	82 kDa	*Cardiovasc Res.* 2006 69(2):491-501
PKCδ	BD Biosciences 610397	Human, rat mouse	Western Blot	1:2000	78 kDa	None.
RhoA	Cell Signaling 67B9	Human, monky	Western Blot	1:2000	21 kDa	*Cardiovasc Res.* 2006 69(2):491-501
ROCK2	BD Biosciences 610623	Human, rat mouse	Western Blot	1:2000	180 kDa	*J Clin Invest.* 2015 125(1):324-36
SMαA	Sigma-Aldrich A2547	Human, rat mouse	Western Blot	1:5000	42 kDa	*J Biol Chem.* 2012 287(29):24739-53
Sp1	Santa Cruz Biotech sc-59	Human, rat mouse	Western blot ChIP, EMSA	1:2000 1-2 μg	81 kDa	*J Biol Chem.* 2012 287(14):10799-811
Sp1	Cell Signaling 9389	Human, rat mouse	DPI-ELISA	1:1000	81 kDa	None
TNF	IHC world, IW-PA1122	Human, rat mouse	IHC	Working solution	22 kDa	*J Biol Chem.* 2012 287(29):24739-53

Table 2. PCR primers & probes

Gene	Primer	Sequence	Application
Rat CPI-17	Forward	5'-TCGACGGACGCTTGGAA-3'	Real-time PCR
	Reverse	5'-CAGCAGCTCGTCGATGTTGA-3'	
Mouse PDGF-B	Forward	5'-GTAATCGCCGAGTGCAAGA-3'	Real-time PCR
	Reverse	5'-CGTTGGTGCGATCGATGA-3'	
Mouse CPI-17	Forward	5'-CTAGCTAGCTTCTCTACTCTCTCCTTCTCTCT-3'	Cloning -792 promoter
	Reverse	5'-CCCAAGCTTATCGTACCGTCACCCGC-3'	
Mouse CPI-17	Forward	5'-CTAGCTAGCATCGCAACAGGAACAAGTGA-3'	Cloning -592 promoter
	Reverse	5'-CCCAAGCTTATCGTACCGTCACCCGC-3'	
Mouse CPI-17	Forward	5'-CTAGCTAGCACAGGAGGTTTAGGAGTTTAAGGC-3'	Cloning -392 promoter
	Reverse	5'-CCCAAGCTTATCGTACCGTCACCCGC-3'	
Mouse CPI-17	Forward	5'-CTAGCTAGCTCTAAGATGGGCACCCCG-3'	Cloning -203 promoter
	Reverse	5'-CCCAAGCTTATCGTACCGTCACCCGC-3'	
Mouse CPI-17	Forward	5'-GCTAGCTCGGGGAGGGGCGGG-3'	Cloning -92 promoter
	Reverse	5'-CCCAAGCTTATCGTACCGTCACCCGC-3'	
Mouse CPI-17	Forward	5'-TCCCGGAGCGCGAGGCAGGGCGCAAAGTAGGGGCAG CGGGTGACGGTACGAT-3'	Cloning -27 promoter
	Reverse	5'-ATCGTACCGTCACCCGCTGCCCCTACTTTGCGCCCTG CCTCGCGCTCCGGGA-3'	
Mouse CPI-17	Forward	5'-GGCGATCGGGGAGGGAAAGGTCCCAGAGCACGA-3'	GC-box 1 mutation & EMSA
	Reverse	5'-TCGTGCTCTGGGACCTTTCCCTCCCCGATCGCC-3 '	
Mouse CPI-17	Forward	5'-GGTCCCAGAGCACGAGCCCCAAACCCCGGTGGCCCC-3'	GC-box 2 Mutation & EMSA
	Reverse	5'-GGGGCCACCGGGGTTTGGGGCTCGTGCTCTGGGACC-3'	
Mouse CPI-17	Forward	5'-CGCCCCCGGTGGCCCAAACCCGGCCATTAAGAT-3'	GC-box 3 Mutation & EMSA
	Reverse	5'-ATCTTAATGGCCGGGTTTGGGCCACCGGGGGCG-3'	
Mouse CPI-17	Forward	5'-CTCTTAAAAAGAGAAACTTCAGTCTCATCCCCCTC CCGT ACA GCT-3'	NFκB mutation
	Reverse	5'-AGCTGTACGGGAGGGGGATGAGACTGAAGTTTCT CTTTTTAAGAG-3'	

Supplemental Table 2. PCR primers & probes (continued)

Gene	Primer	Sequence	Application
Mouse CPI-17	Forward	5’-TGCACATTAGCCAGCTTGC-3’	SP1/KLF4 ChIP
	Reverse	5’-ATCGTACCGTCACCCGCT-3’	
Mouse CPI-17	Forward	5’-CTGGCAGCAGCCAGGTAT-3’	Control ChIP
	Reverse	5’-GAGTAGAGAAGTAGAGGCCGGC-3’	
Mouse CPI-17	Forward	5’-GGCGATCGGGGAGGGGCGGGTCCCAGAGCACGA-3’	EMSA GC-box 1
	Reverse	5’-TCGTGCTCTGGGACCCGCCCCTCCCCGATCGCC-3’	
Mouse CPI-17	Forward	5’-GGTCCCAGAGCACGAGCCCCCGCCCCCGGTGGCCCC-3’	EMSA GC-box 2 & DPI-ELISA
	Reverse	5’-GGGGCCACCGGGGGCGGGGGCTCGTGCTCTGGGACC-3’	
Mouse CPI-17	Forward	5’-CGCCCCCGGTGGCCCCGCCCCGGCCATTAAGAT-3’	EMSA GC-box 3
	Reverse	5’-ATCTTAATGGCCGGGGCGGGGCCACCGGGGGCG-3’	
Rabbit CPI-17	Forward	5’-CTGGACGTGGAGAAGTGGAT-3’	Real-time PCR
	Reverse	5’-CTCCTGGACGAAGTCCTCTG-3’	
